# Correlation of clinical parameters with endolymphatic hydrops on MRI in Meniere's disease

**DOI:** 10.3389/fneur.2022.937703

**Published:** 2022-07-25

**Authors:** Seung Cheol Han, Young Seok Kim, Yehree Kim, Sang-Yeon Lee, Jae-Jin Song, Byung Yoon Choi, Ji-Soo Kim, Yun Jung Bae, Ja-Won Koo

**Affiliations:** ^1^Department of Otorhinolaryngology, Seoul National University College of Medicine, Seoul National University Bundang Hospital, Seongnam, South Korea; ^2^Department of Otorhinolaryngology, Seoul National University College of Medicine, Seoul National University Hospital, Seoul, South Korea; ^3^Department of Neurology, Seoul National University College of Medicine, Seoul National University Bundang Hospital, Seongnam, South Korea; ^4^Department of Radiology, Seoul National University College of Medicine, Seoul National University Bundang Hospital, Seongnam, South Korea; ^5^Sensory Organ Research Institute, Seoul National University Medical Research Center, Seoul, South Korea

**Keywords:** Meniere disease, endolymphatic hydrops, labyrinth diseases, magnetic resonance imaging, clinical features

## Abstract

A clinical diagnosis of Ménière's disease (MD) is made based on medical history and audiometry findings. The 1995 American Academy of Otolaryngology-Head and Neck Surgery (AAO-HNS) guidelines requires histopathological confirmation of endolymphatic hydrops (EH) for a diagnosis of “certain” MD. Symptoms such as dizziness and ear fullness are important diagnostic features; however, the descriptions provided by patients are frequently vague and non-specific. A recently developed magnetic resonance imaging (MRI) protocol to document EH is, therefore, useful for the evaluation of inner ear status in patients with MD. In this study, patients with MD were assessed using MRI and the HYDROPS (HYbriD of Reversed image Of Positive endolymph signal and native image of positive perilymph Signal) protocol to investigate the effectiveness of MRI for visualization of the endolymphatic space in the diagnosis of MD by correlating clinical laboratory parameters with the grade of EH. Of the 123 patients with MD recruited in this study, 80 had definite MD, 11 had probable MD, and 32 had possible MD based on the 1995 AAO-HNS guidelines. The EH grade based on HYDROPS MRI was determined independently by two otorhinolaryngologists and compared with several clinical parameters, including the diagnostic scale of MD (1995 AAO-HNS guidelines), pure tone average (PTA), low tone average (LTA), canal paresis (CP) on the caloric test, and disease duration. Cochlear hydrops and vestibular hydrops were detected in 58 and 80% of 80 definite MD ears, in 33 and 58% of 12 probable MD ears, and in 5 and 27% of 37 possible MD ears, respectively. The proportion of higher hydrops grades increased significantly with grade according to the MD diagnostic scale (*p* < 0.0001). Both PTA and LTA were significantly higher in patients with hydrops grade 2 than hydrops grade 0 in both the cochlea and the vestibule. CP was significantly higher in patients with grade 2 than grade 0 vestibular hydrops. Disease duration was not associated with hydrops grade. Radiological evaluation of MD using the HYDROPS protocol is useful for evaluation of the extent and severity of EH in the diagnosis of MD based on its pathophysiological mechanism.

## Introduction

Ménière's disease (MD) is a multifactorial disorder characterized by recurrent vertigo, fluctuating hearing change, ear fullness, and tinnitus. According to the 1995 Hearing and Equilibrium guidelines of the American Academy of Otorhinolaryngology-Head and Neck Surgery Committee (AAO-HNS), histopathological confirmation of endolymphatic hydrops (EH) is required for a diagnosis of “certain” MD. However, diagnosis of “certain” MD is practically impossible in living patients (1).

Some patients with cochleovestibular symptoms but who do not satisfy the criteria of the MD diagnostic guidelines may have EH, which is universally recognized as a component of MD pathogenesis (2–5). In contrast, not all patients with clinically diagnosed MD have endolymphatic hydrops (6–9). Furthermore, EH may be asymptomatic; when suspected, it must be distinguished from other otological disorders, such as inner ear trauma (1), viral infection, autoimmune processes (10), and cellular channelopathies (11).

A number of techniques have been developed to visualize EH using magnetic resonance imaging (MRI). In 2007, MRI visualization of the inner ear was facilitated by the intravenous (IV) injection of gadolinium-based contrast material (Gd) (12), rather than intratympanic (IT) injection (13). In MRI of the inner ear, despite the 4-h delay after injection, IV-Gd is faster and much less invasive than IT-Gd (14, 15). However, unlike MRI using IT-Gd, IV-Gd does not permit 3D inversion recovery sequences with a “real” reconstruction protocol (3D-real IR protocol) (16); the lower concentration of Gd in such sequences enables separate visualization of endolymph, perilymph, and bone in a single image. The recently developed “HYDROPS” (HYbriD of the reversed image of positive endolymph signal and native image of positive perilymph signal) imaging protocol addresses this limitation by enabling recognition of the endolymphatic space (17).

Further advances have yielded HYDROPS2, HYDROPS-Mi2, and HYDROPS2-Mi2, which use MR cisternography for better contrast in the production of positive perilymph images (PPIs) and positive endolymph images (PEIs) (18). Combined with the ratio of the endolymphatic space to the entire lymphatic space (%EL), the vestibular hydrops ratio (%VH), or the relative vestibular hydrops ratio (%RVH), these perilymph and endolymph images have been used in attempts to identify inner ear MRI findings associated with the clinical parameters of MD (19, 20). However, most studies have been performed only on small population of patients with definite MD who already have severe symptoms. In addition, it can be inferred that these patients have higher rates of severe EH. Therefore, the correlation between the clinical characteristics of MD and EH from MRI findings should be examined depending on the diagnostic scale of MD (possible, probable, and definite) and even unaffected subjects, determining the wider correlations between the severity of EH and clinical characteristics of patients with MD.

In this study, patients with clinically diagnosed MD defined according to the diagnostic scale of the 1995 AAO-HNS guidelines were examined by HYDROPS MRI. The correlations of EH grades based on HYDROPS MRI (21) with clinical and laboratory parameters were examined.

## Materials and methods

### Study setting and patients

HYDROPS MRI was performed in 166 patients with recurrent vertigo and hearing problems between 1 January 2020 and 31 August 2021. Detailed history taking, neurotological evaluation, and audiovestibular laboratory tests were performed. Forty-three patients were excluded due to tumors in the cerebellopontine angle, internal auditory canal, or inner ear; superior canal dehiscence syndrome; or recurrent dizziness that did not meet the diagnostic criteria of MD (1995 AAO-HNS guidelines). Finally, 123 patients (49 men, 74 women, mean age of 58.9 years) were included in this study. Based on the 1995 AAO-HNS guidelines, 32 patients had possible MD, 11 had probable MD, and 80 had definite MD. The left side was involved in 68 patients, while the right side was involved in 49 patients. Of the remaining six patients with bilateral MD, five had possible MD and one had probable MD. Among the 117 patients with unilateral MD, the contralateral side was clinically unaffected. Therefore, based on the MD classification, there were 117 unaffected ears and 129 affected (37 possible, 12 probable, and 80 definites) ears (Table 1). This study was approved by the review board of the Clinical Research Institute at Seoul National Bundang Hospital and was conducted in accordance with the Declaration of Helsinki (IRB-B-2111-720-106).

**Table 1:** Demographic characteristics of 123 patients diagnosed with Ménière's disease.

**Sex (male:female)**	**49:74**
Age (years)	58.96 ± 13.63
Affected side (right:left:bilateral)	49:68:6
Unaffected:affected ears	117:129
Disease classification (definite:probable:possible)	80:11:32

### Inner ear MRI

MRI was performed using a 3.0 Tesla machine (Ingenia CX; Philips, Amsterdam, The Netherlands). MR scanning was performed 4 h after IV injection of Gadobutrol (Gadovist; Bayer, Leverkusen, Germany) (0.1 mmol/kg body weight).

Heavily T2-weighted MR cisternography (MRC) was performed for the anatomical reference of the total lymph fluid. Heavily T2-weighted-3D-FLAIR (hT2W-3D-FLAIR) scan with a repetition time (TR) of 16,000 ms was performed, with an inversion time of 2,900 ms at 4 h after IV injection of Gadobutrol in accordance with a previous report (22) and to distinguish PPI from other images. The hT2W-3D-FLAIR scan was performed with the same TR and inversion time of 2,000 ms at 4 h after IV injection of Gadobutrol to distinguish PEI. The perilymph signal is increased in images with an inversion time of 2,900 ms and suppressed in images with an inversion time of 2,000 ms. The endolymph signal is increased in images with an inversion time of 2,000 ms. The HYDROPS image was obtained by subtracting PEI from PPI at the scanner console. Negative signal values were allowed for the subtraction.

Detailed parameters for PPI and PEI were as follows: FLAIR–volume isotropic turbo spin-echo acquisition sequence; repetition time, 16,000 ms; echo time, 544 ms; echo train length, 173; matrix size, 332 × 328 mm; slice thickness, 1.6 mm; field of view, 200 × 200 mm; sensitivity encoding acceleration factor, two; number of excitations, two; scan time, 3 min 45 s.

### Image analysis

Two otorhinolaryngologists blinded to the patient's clinical information, including disease site and history, independently rated the hydrops grade of the cochlea and vestibule using the grading system proposed by Nakashima et al. (21).

**Figure 1:**
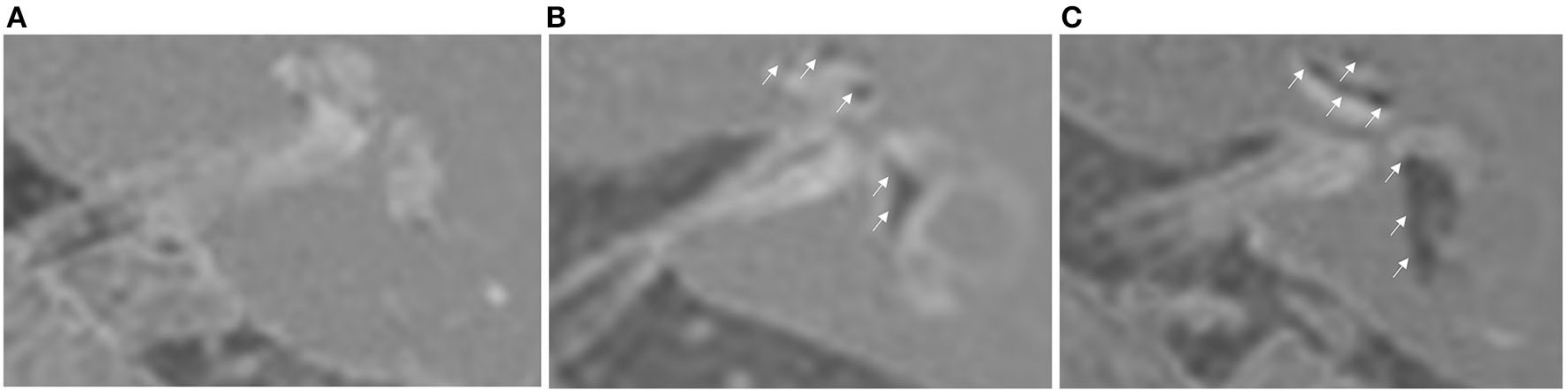
Hydrops grading based on HYDROPS MRI of the left ear. **(A)** Grade 0 cochlear hydrops with grade 0 vestibular hydrops. **(B)** Grade 1 cochlear hydrops with grade 1 vestibular hydrops. **(C)** Grade 2 cochlear hydrops with grade 2 vestibular hydrops.

Briefly, in the cochlea, displacement of Reissner's membrane was defined as EH. Patients in whom the area of the cochlear duct exceeded the area of the scala vestibuli were diagnosed with significant hydrops. If the grade of EH differed between the basal and upper turns, a higher grade of EH was reported. In the vestibule, EH was defined as a ratio of the area of the endolymphatic space to that of the vestibular fluid space exceeding 1/3. Patients in whom the endolymphatic space was >50% of the fluid area in the vestibule were diagnosed with significant hydrops. Grade 0 was defined as no EH, grade 1 as mild EH, and grade 2 as significant EH (21) (Figure 1).

Interrater reliability analysis was performed between the two otorhinolaryngologists before reaching a consensus on the hydrops grading results. Cohen's kappa value was 0.870, indicating good agreement between the two otorhinolaryngologists. Grade rating was different in 6 ears (cochlea) and 25 ears (vestibule) by 1 grade. The final grade was determined after repeating the rating of these images by a third radiologist.

### Clinical parameters

The diagnostic scale of the 1995 AAO-HNS guidelines was used for the analysis of clinical parameters in the diagnosis of MD. Pure tone audiometry and bithermal caloric tests were performed; if the tests were not performed on the day of HYDROPS MRI or their results were unavailable, the results of tests performed closest to the date of HYDROPS MRI were used for the analysis. The disease duration (in months) was calculated from the onset of the first episode of vertigo to the date of HYDROPS MRI confirmation.

All 123 patients underwent a hearing test using pure tone audiometry. Threshold results were obtained at frequencies of 0.25, 0.5, 1, 2, 3, 4, and 8 kHz in a soundproof audio booth. The pure tone average (PTA) was calculated based on the average of audiometry thresholds at 0.5, 1, 2, and 3 kHz according to the 2008 American Medical Association method for the estimation of hearing disability. The low tone average (LTA) was also calculated based on the average of the audiometry thresholds at 0.25, 0.5, and 1 kHz.

Bithermal caloric test results were available for 107 patients. The test was performed using a water caloric stimulator (NCI480; ICS Medical, Schaumburg, IL, USA) with the patient in the supine position and the head elevated at 30°. Caloric irrigation was delivered in the order of right cool (30°C), left cool (30°C), right warm (44°C), and left warm (44°C) for 30 s each, at a flow rate of 300 ml/min. The maximum slow-phase velocity of nystagmus was calculated after irrigation at each temperature; CP was determined using Jongkees' formula (23).

### Statistical analysis

The associations of hydrops grade with PTA, LTA, CP, and disease duration were analyzed by one-way ANOVA with Scheffé's *post-hoc* multiple comparison tests and by Welch's ANOVA with the Games-Howell's *post-hoc* multiple comparison test. The association between hydrops grade and MD classification was analyzed using the Fisher's exact test with *post-hoc* analysis consisting of pairwise Fisher's exact tests. The data were analyzed using R Studio version 1.4.1717 (R Studio Team, 2021) and R version 3.5.2 (R Core Team, 2018). In all analyses, *p* < 0.05 was taken to indicate statistical significance.

## Results

### Comparison of hydrops grade based on the diagnostic scale of MD

Of the total of 246 ears, 42 (17.1%) and 14 (5.7%) ears showed grade 1 and grade 2 cochlear hydrops, respectively, and 55 (22.4%) and 41 (16.7%) ears showed grade 1 and grade 2 vestibular hydrops, respectively. There were no hydropic changes (grade 0) in 190 ears (77.2%) for the cochlea and in 150 ears (60.9%) for the vestibule. Seven ears (2.8%) had only cochlear hydrops, and 47 (19.1%) ears had only vestibular hydrops. Both cochlear and vestibular hydrops were observed in 49 ears (19.9%).

**Figure 2:**
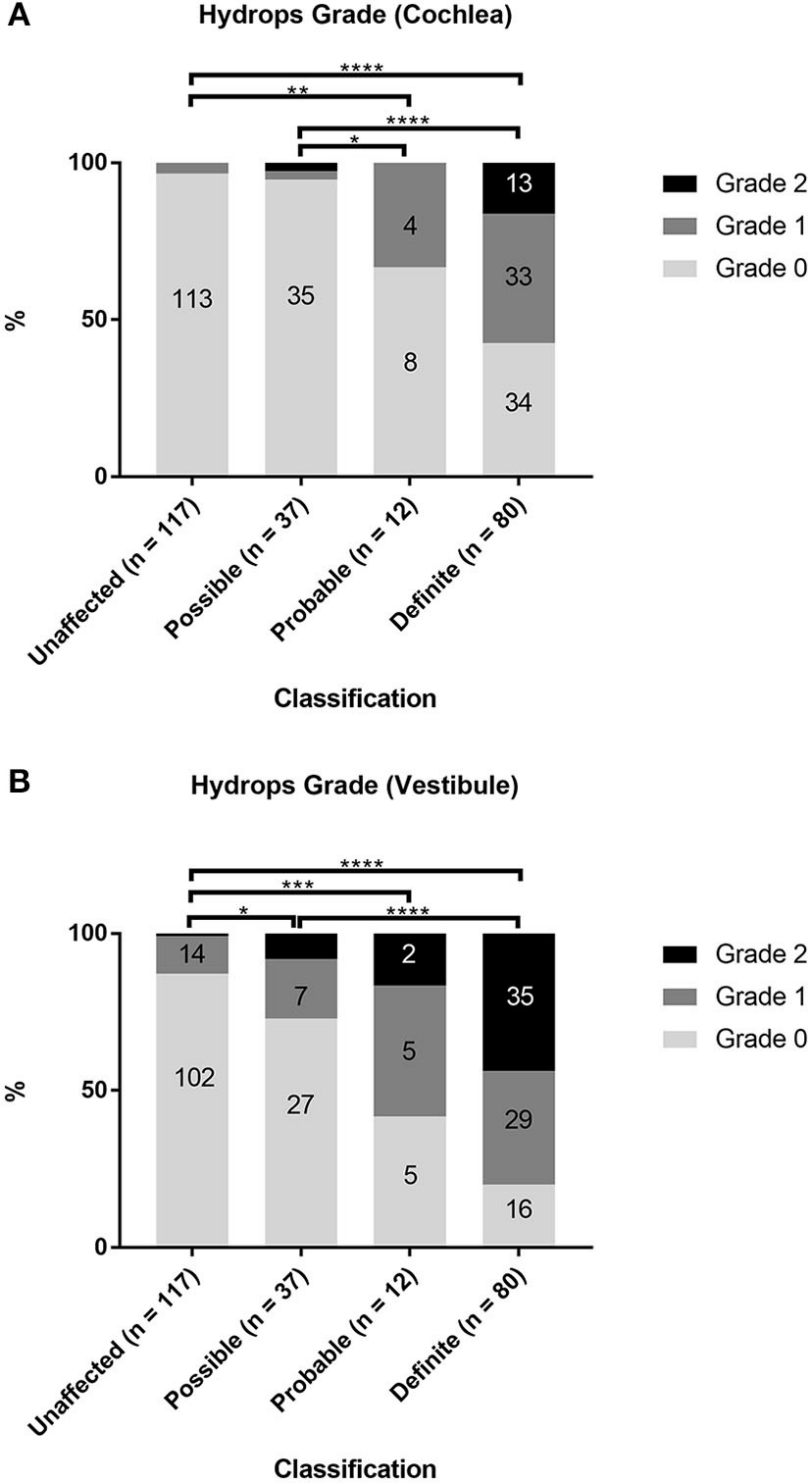
Comparison of hydrops grade according to the diagnosis of Ménière's disease (MD). The proportions of hydrops grades 0, 1, and 2 in unaffected ears and in ears with possible, probable, or definite MD were evaluated separately in the cochlea **(A)** and vestibule **(B)**. Fisher's exact test with pairwise Fisher's exact tests as *post-hoc* analysis. *p < 0.05; **p < 0.01; ***p < 0.001; ****p < 0.0001.

The associations between the diagnostic scale of MD (unaffected, possible, probable, and definite) and the hydrops grade in the cochlea and vestibule were examined in 246 ears of 123 patients (Supplementary Table 1; Figure 2). Among the cases of definite MD, 46 of 80 ears (57.5%) showed grade 1 or 2 cochlear hydrops, and 64 ears (80%) showed grade 1 or 2 vestibular hydrops. Among the cases of probable MD, 4 of 12 ears (33%) showed grade 1 cochlear hydrops, and seven ears (58.3%) showed grade 1 or 2 vestibular hydrops. Among the cases of possible MD, two of 37 ears (5.4%) showed grade 1 or 2 cochlear hydrops, and 10 ears (27%) showed grade 1 or 2 vestibular hydrops. Among the 117 unaffected ears, 4 (3.4%) showed grade 1 cochlear hydrops and 15 (12.8%) showed grade 1 or 2 vestibular hydrops. Statistical analyses using Fisher's exact test showed that hydrops grade proportions were significantly different according to the diagnostic scale of MD both in the cochlea (*p* < 0.0001) and in the vestibule (*p* < 0.0001) (Supplementary Tables 2, 3). The proportions of hydrops grades 1 and 2 increased, while the proportion of hydrops grade 0 decreased as the diagnostic scale of MD increased in the order unaffected, possible, probable, and definite (Figure 2). The results of *post-hoc* analysis suggested that, for cochlear hydrops grade, the definite and probable MD groups differed significantly from the unaffected and possible MD groups (Supplementary Table 2). For the vestibular hydrops grade, the unaffected group differed significantly from all affected groups, and the possible group differed significantly from the definite group (Supplementary Table 3).

### Comparison of hearing thresholds according to hydrops grade detected by MRI

**Figure 3:**
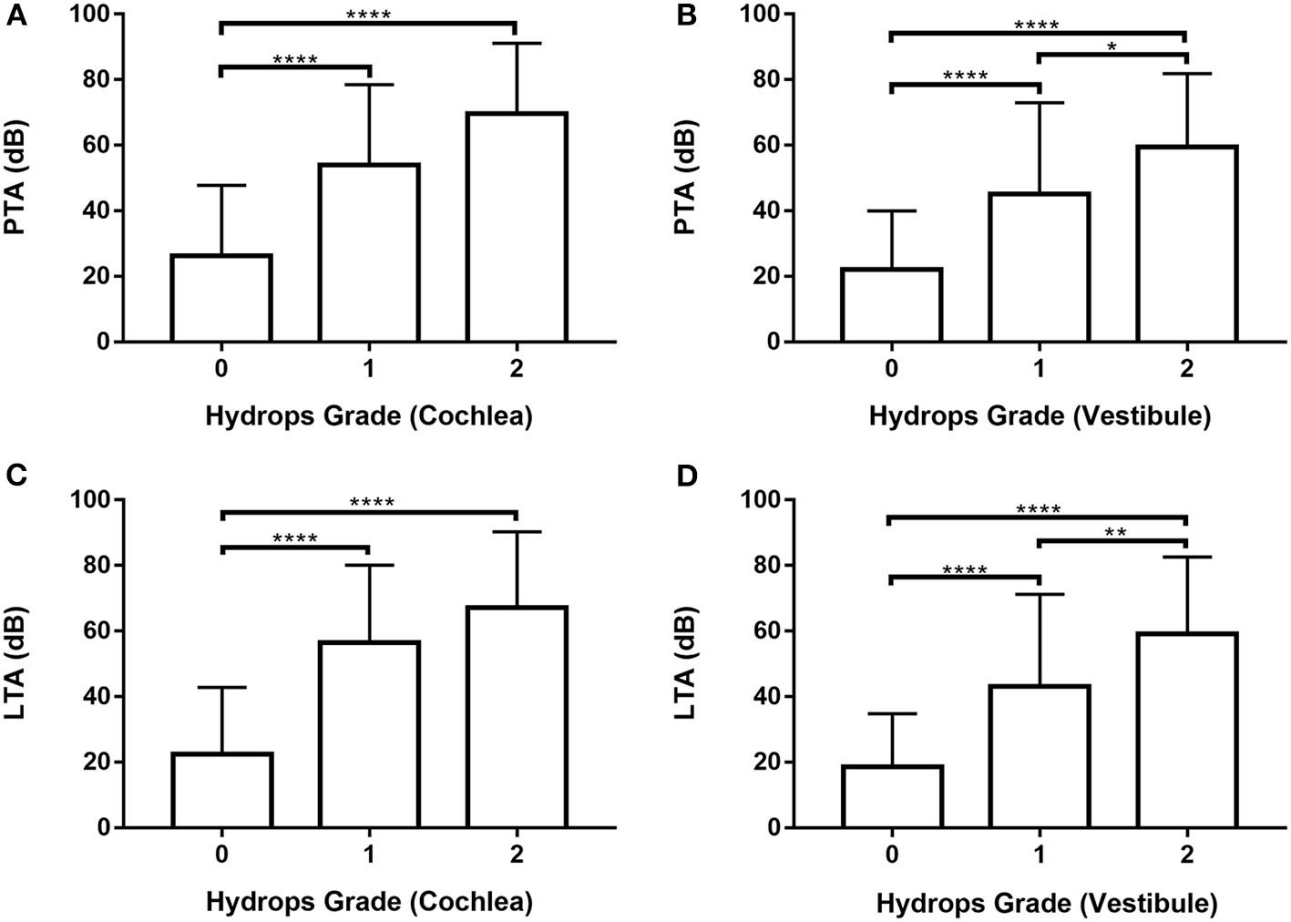
Analysis of pure tone average (PTA) and low tone average (LTA) according to cochlear and vestibular hydrops grades (0–2) in 246 ears of 123 patients. **(A)** and **(C)** One-way ANOVA with Scheffé's *post-hoc* multiple comparison test. **(B)** and **(D)** Welch's ANOVA with Games-Howell's *post-hoc* multiple comparison test. *p < 0.05; **p < 0.01; ****p < 0.0001.

Audiometry data for all 246 ears were analyzed (Supplementary Tables 4, 5; Figure 3) by one-way ANOVA followed by Scheffé's *post-hoc* multiple comparison tests; they were also analyzed using Welch's ANOVA followed by Games-Howell's *post-hoc* multiple comparison test. PTA and LTA increased significantly in patients with higher vestibular hydrops grades (Figures 3B,D). Both PTA and LTA were significantly lower in patients with grade 0 than grades 1 and 2 cochlear hydrops (i.e., the values were lower in ears without cochlear EH than in ears with cochlear EH) (Figures 3A,C).

### Comparison of hearing thresholds according to the hydrops grade on MRI in ears with definite MD

**Figure 4:**
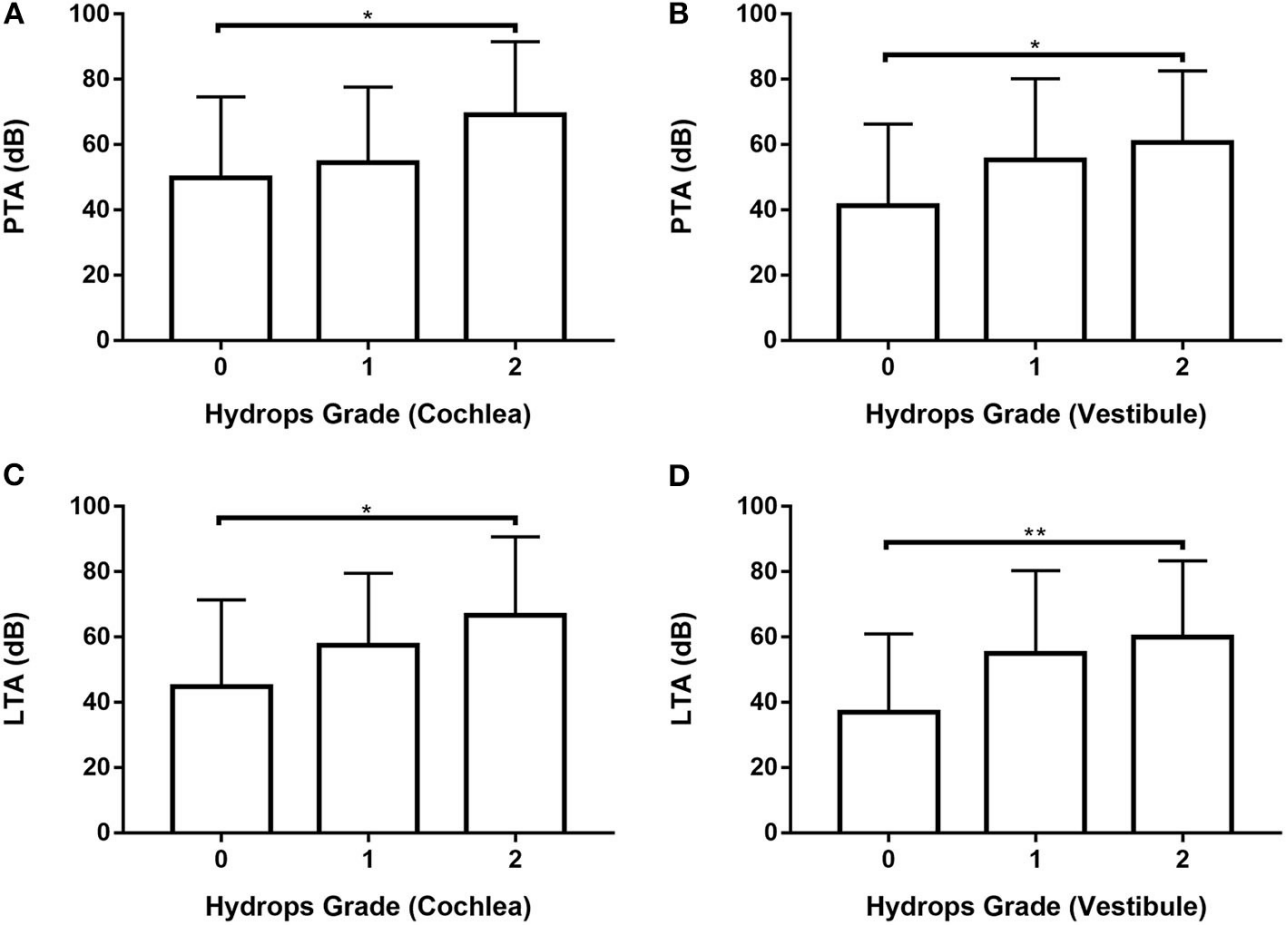
Analysis of PTA (**A**,**B**) and LTA (**C**,**D)** according to hydrops grade in the cochlea (**A**,**C)** and vestibule (**B, D**) in 80 ears with definite MD by one-way ANOVA with Scheffé's *post-hoc* multiple comparison test. *p < 0.05; **p < 0.01.

Data from 80 ears with definite MD were analyzed (Supplementary Tables 6, 7; Figure 4) by one-way ANOVA with the Scheffé's *post-hoc* multiple comparison test. Analysis of both PTA and LTA values indicated that hearing thresholds were significantly higher in patients with hydrops grade 2 than hydrops grade 0 in both the cochlea and the vestibule.

### Comparison of CP according to hydrops grade in MD ears

**Figure 5:**
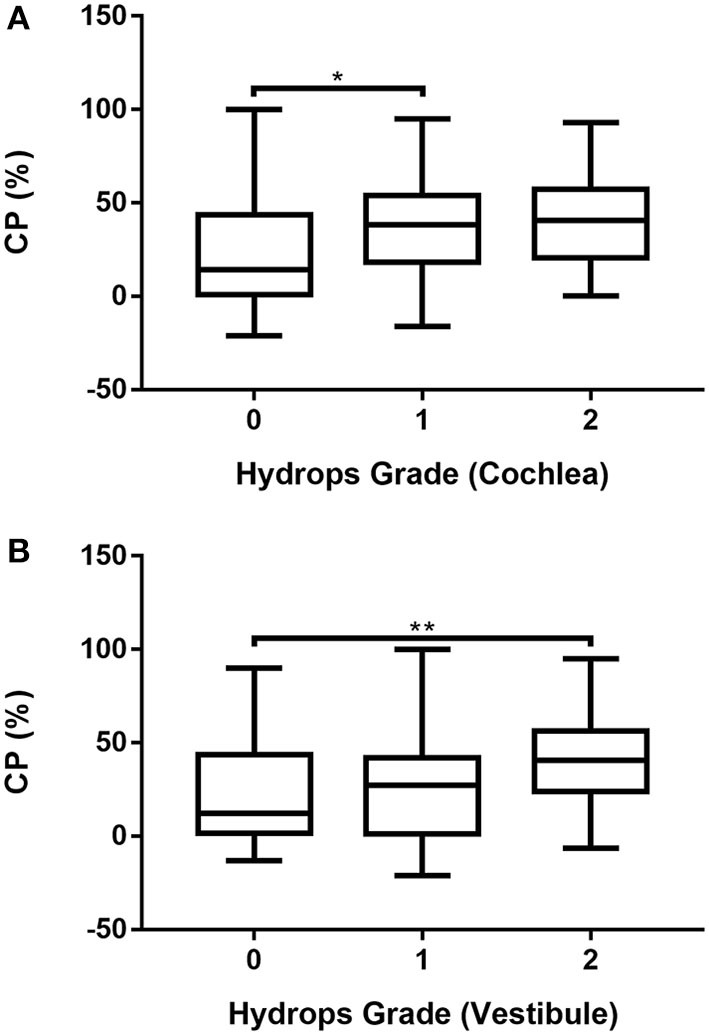
Associations of cochlear **(A)** and vestibular **(B)** hydrops grades 0, 1, and 2 with CP in 107 patients with unilateral MD determined by one-way ANOVA with Scheffé's *post-hoc* multiple comparison test. *p < 0.05; **p < 0.01.

The association between CP and hydrops grade was analyzed after excluding six patients with bilateral MD and 10 patients who refused the caloric test. Accordingly, caloric test results were available for 107 ears. The association between CP and hydrops grade (0–2) in MD ears was analyzed by one-way ANOVA followed by the Scheffé's *post-hoc* multiple comparison test. The results are shown in Supplementary Table 8 and Figure 5. CP was significantly smaller in ears with grade 0 cochlear hydrops than in those with grade 1 cochlear hydrops (Supplementary Table 8; Figure 5A) and was significantly greater in ears with grade 2 vestibular hydrops than in those with grade 0 vestibular hydrops (Supplementary Table 8; Figure 5B).

### Disease duration and hydrops grade

The association between disease duration and hydrops grade was analyzed in the ears of all 123 patients with MD (Supplementary Table 9; Supplementary Figures 1A,B). The results of one-way ANOVA followed by the Scheffé's *post-hoc* multiple comparison test showed no significant association between disease duration and hydrops grade.

## Discussion

In this study, the hydrops grade of the cochlea and the vestibule was examined in 246 ears of 123 patients. Cochlear hydrops and vestibular hydrops were detected in 58 and 80% of 80 definite MD ears, respectively, in 33 and 58% of 12 probable MD ears, and in 5 and 27% of 37 possible MD ears, respectively. The proportion of a higher hydrops grade increased significantly with increasing grade of MD classification (unaffected, possible, probable, and definite). The higher proportion of positive hydrops in definite MD suggested that EH is the pathophysiological mechanism of MD.

However, 3% of the cochleae and 13% of the vestibules of 117 unaffected ears also showed hydrops in this study. This is not surprising because varied rates of EH in unaffected ears have been reported in the literature. In a study conducted on a population of 29 patients with definite unilateral MD, 48 and 55% showed EH in the cochlea and the vestibule, respectively, in unaffected ears (24). In contrast, Wu et al. reported that 16.7% of 54 patients with unilateral definite MD revealed EH in the cochlea and/or vestibule (25). The possibility of subclinical involvement of the contralateral ear in patients with MD was not only documented in imaging studies using HYDROPS MRI but also demonstrated in an electrophysiological study. In a study to test the vestibular-evoked myogenic potential (VEMP) in unilateral patients with MD, unaffected MD ears also showed a similar pattern to affected MD ears, such as elevated thresholds and frequency tuning around 1 kHz, unlike normal controls (26). These observations constitute evidence of subclinical involvement in the contralateral ear in unilateral MD. However, the incidence of EH in unaffected ears in our study was relatively low. This can be explained by the likely inclusion of patients with possible and probable MD in our study. As unaffected ears in MD may also have the potential to develop MD, it would be interesting to determine whether radiological EH without clinical symptoms (silent EH) precedes and eventually progresses to clinical EH.

Patients with clinically suspected MD, such as those with probable and possible MD, showed much lower incidences of EH. In probable MD, 33% showed cochlear hydrops and 58% showed vestibular hydrops. In possible MD, 5 and 27% of cases showed cochlear and vestibular hydrops, respectively. By adopting the 1995 AAO-HNS-defined definite, probable, and possible MD diagnostic scale (1) instead of the criteria of the 2015 Barany Society guidelines (27), we could learn the proportion of the patients having EH depending on the different diagnostic scales of MD since MD shows a wide spectrum of clinical presentations. True attacks of MD can have different visual analog scale (VAS) ranges and various hearing changes. There are also adjuvant spells of dizziness with different severities between true attacks of MD. In the 1995 AAO-HNS diagnostic scale (1), definite MD and certain MD are distinguished, which is now possible by the development of MRI protocol to visualize the endolymphatic space. It was interesting that 57 and 80% of definite MD ears showed hydrops in the cochlea or the vestibule, respectively, and those ears can be diagnosed as “certain” MD.

Using the 1995 AAO-HNS diagnostic scale (1), we can diagnose patients with the first attack of MD with low-frequency hearing loss, as probable MD, and patients with mild or moderate sensorineural hearing loss with flat audiometry results, as definite, probable, or possible MD depending on the pattern of the vertigo attacks. In this way, we could learn the proportion and the severity of hydrops in patients with the first attack of MD and patients with possible MD. In the 2015 Barany Society guidelines (27), these patients are not considered as MD even though they have recurrent inner ear symptoms and EH on MRI. Therefore, the 1995 AAO-HNS diagnostic scale (1) was more appropriate in our study to understand the underlying pathophysiology of this disease with a wide spectrum of clinical presentations. However, there may be some controversy regarding this point, so our choice of the 1995 AAO-HNS diagnostic scale (1) instead of the criteria of the 2015 Barany Society guidelines (27) as the main guidelines may represent a limitation of this study. The size of the study population consisting of 123 (246 ears) in our study was much larger than the sample sizes in previous studies of the associations between clinical parameters of MD and EH visualized by MRI (19, 20, 28–33).

In addition to a history of episodic vertigo with documented sensorineural hearing loss, inner ear MRI may substitute for histopathological evaluation by demonstrating EH to meet the “certain” classification of MD as defined by the AAO-HNS diagnostic scale. In our study, the endolymphatic space (as visualized on MRI using the HYDROPS protocol) was significantly expanded in patients with MD; EH grade on HYDROPS MRI exhibited associations with disease classification and clinical laboratory parameters. These results demonstrated the clinical importance of EH visualization in patients with MD using the HYDROPS MRI protocol.

Previous histopathological studies in postmortem samples of the temporal bones showed that, in the early stage of MD, hydrops predominantly involves the cochlear duct and saccule, while the utricle, ampulla, and overall endolymphatic system are affected in later stages (34–36). This sequence may reflect the mechanical compliance of the labyrinth membrane, which is higher in the saccule than the utricle (36, 37). In addition, the thick amorphous material in the ampulla and semicircular canal endows the labyrinth membrane with greater mechanical resistance (36). A relationship has been identified between the histological degree of cochlear involvement and the extent of hearing loss, which is more consistent than the relationship between caloric test responses and histologically confirmed dilation of the vestibular structure (9, 38). Accordingly, the degree of hydrops is greater and involves a wider area in patients with severe MD, consistent with the analysis of the proportions of hydrops with respect to the MD classification.

Several recent studies analyzed MRI findings of EH and clinical data (19, 20, 28–31). Two Korean studies calculated the mean hydrops ratios in study populations of 29 and 16 patients with unilateral definite MD (19, 20). The mean hydrops ratios were 0.372 and 0.667 for the cochlea and the vestibule in one study (19) and 0.725 for the vestibule in the other study (20). Both studies analyzed correlations between the severity of EH and laboratory parameters, including audiometry, caloric test, cervical VEMP, and electrocochleography (ECoG), and showed that audiometry and caloric test results were correlated with the hydrops ratio, while VEMP and ECoG were not (19, 20). Other studies have also examined the correlation of EH with cervical VEMP in patients with unilateral definite MD (28, 29). In these studies, EH on MRI was present in 10 of 14 ears (29) and 12 of 20 ears (28), and hydrops grade (28) was correlated with the amplitude of cervical VEMP. In another study, in a population of 78 patients with unilateral definite MD, there were 57 cases of cochlear EH and 60 of vestibular EH, and these were related to the results of audiometry and caloric tests (30). In a study in which MRI was performed before the endolymphatic duct blockage procedure for unilateral definite MD, all affected ears had cochlear and vestibular EH (31).

MRI assessment of EH severity is usually based on the hydrops ratio or grade. Assessment based on the hydrops ratio uses the vestibular endolymphatic ratio, the cochlear endolymphatic ratio (32), and the cochlear hydrops ratio, along with the vestibular hydrops ratio (19, 20, 31) and the ratio of the area of the endolymphatic space to that of the entire lymphatic space (19). These ratios are obtained by drawing the region of interest for a specific slice of the MRI data and then calculating the number of pixels. Although an automated method for measurement of the hydrops ratio from the MRI findings was recently reported and showed considerable agreement with the existing calculation method, this automated measurement is not easy to set up for general clinical practice (39). Instead, the classification described by Nakashima et al. (21) can be easily adapted to determine the EH grade in daily clinical practice (29, 31–33). The grading systems proposed by Barath et al. (15), Kahn et al. (40), and Bernaerts et al. (41) have also been adopted for the analysis of EH in MD (28, 30); in some instances, these systems cannot be combined with MRI using the HYDROPS protocol. Therefore, the simplified grading system proposed by Nakashima et al. (21) was used in this study. Further advantages of this method include its ease of use for clinicians, as well as the absence of a requirement for another program or additional work for calculations. However, this grading system is subjective because it does not objectively determine the hydrops ratio.

Analysis of 80 ears with definite MD showed that both PTA and LTA were significantly higher in patients with hydrops grade 2 than grade 0 in both the cochlea and the vestibule. CP was significantly higher in patients with grade 2 vestibular hydrops than in those with grade 0 vestibular hydrops. Disease duration was not significantly associated with hydrops grade. These results were comparable with those of many previous studies. Audiometry results are closely associated with EH severity, assessed by grade or ratio (19, 29–32). The PTA results were significantly associated with the presence (19, 29) and severity (31, 32) of EH on MRI, as were the LTA results (19, 30, 32). In this study, we also examined the associations of vestibular hydrops grades 0, 1, and 2 with the PTA and LTA results in ears unaffected by MD, as well as their associations with possible, probable, and definite MD. Our results showed that in patients with MD, regardless of the classification, the presence of hydrops was significantly associated with the PTA and LTA results in both the cochlea and the vestibule. PTA and LTA findings seem to predict EH more accurately in definite MD.

There have been varied results regarding the correlation between radiological EH and the results of the caloric test, with some studies failing to show a relationship between radiological EH and CP (30, 32), while others have reported significant correlations (19, 20). The CP results were significantly correlated with the degree of EH in this study. We postulated that the correlation between cochlear hydrops and CP may be due to the confounding effect of cochlear hydrops and vestibular hydrops. The Cramer's V between cochlear hydrops grade and vestibular hydrops grade was 0.435 in 107 ears included in the analysis of CP. Therefore, the correlation between cochlear hydrops and CP may be affected by the correlation between vestibular hydrops and CP.

The disease duration and the severity of EH were not correlated with each other in this study. As the disease duration is defined as the period from the first occurrence of MD symptoms to the time of MRI, the severity of cochleovestibular dysfunction seems to be more closely related to the severity of EH than disease duration.

## Conclusion

In our study, the EH grade, as visualized on HYDROPS MRI, was significantly associated with the diagnostic scale of MD according to the 1995 AAO-HNS guideline (1) and was also associated with the PTA, LTA, and CP results. However, the EH grade was not associated with disease duration. These results were obtained in the analysis of unaffected, possible, probable, and definite MD ears. Radiological assessment of EH using HYDROPS MRI is valuable for evaluating the extent and severity of MD, as well as the pathophysiological relationship of MD with EH.

## Data availability statement

The original contributions presented in the study are included in the article/Supplementary Material, further inquiries can be directed to the corresponding author/s.

## Ethics statement

The studies involving human participants were reviewed and approved by Clinical Research Institute at Seoul National Bundang Hospital. The patients/participants provided their written informed consent to participate in this study. Written informed consent was obtained from the individual(s) for the publication of any potentially identifiable images or data included in this article.

## Author contributions

SH and J-WK designed this study and wrote the manuscript. SH, YoK, YeK, and J-WK collected and analyzed data. All authors contributed to the article and approved the submitted version.

## Funding

This study was supported by the Seoul National University Bundang Hospital Grant number (2021-019386).

## Conflicts of interest

The authors declare that the research was conducted in the absence of any commercial or financial relationships that could be construed as a potential conflict of interest.

## Publisher's note

All claims expressed in this article are solely those of the authors and do not necessarily represent those of their affiliated organizations, or those of the publisher, the editors and the reviewers. Any product that may be evaluated in this article, or claim that may be made by its manufacturer, is not guaranteed or endorsed by the publisher.

## References

[1] Committee on Hearing and Equilibrium guidelines for the diagnosis and evaluation of therapy in Meniere's disease. American Academy of Otolaryngology-Head and Neck Foundation, Inc. Otolaryngol Head Neck Surg. (1995) 113:181–5. 10.1016/S0194-5998(95)70102-8 7675476

[2] Chen X Zhang XD Gu X Fang ZM Zhang R. Endolymphatic space imaging in idiopathic sudden sensorineural hearing loss with vertigo. Laryngoscope. (2012) 122:2265–8. 10.1002/lary.23452 22996668

[3] Kasai S Teranishi M Katayama N Sugiura M Nakata S Sone M. Endolymphatic space imaging in patients with delayed endolymphatic hydrops. Acta Otolaryngol. (2009) 129:1169–74. 10.3109/00016480802691143 19863306

[4] Kato M Sugiura M Shimono M Yoshida T Otake H Kato K. Endolymphatic hydrops revealed by magnetic resonance imaging in patients with atypical Meniere's disease. Acta Otolaryngol. (2013) 133:123–9. 10.3109/00016489.2012.726374 23106485

[5] Miyagawa M Fukuoka H Tsukada K Oguchi T Takumi Y Sugiura M. Endolymphatic hydrops and therapeutic effects are visualized in ‘atypical' Meniere's disease. Acta Otolaryngol. (2009) 129:1326–9. 10.3109/00016480802593513 19863332

[6] Berggren S. Histological investigation of three cases with Menie‘re's syndrome. Arch Otolaryngol. (1949) 37:30–6. 10.3109/00016484909120212

[7] Arnvig J. Histological findings in a case of Menie‘re's disease, with remarks on the pathologic-anatomical basis of this lesion. Acta Oto-Laryngologica. (1947) 35:453–66. 10.3109/00016484709123760

[8] Belal A Jr Ylikoski J. Pathologic significance of Meniere's symptom complex. A histopathologic and electron microscopic study. Am J Otolaryngol. (1980) 1:275–84. 10.1016/S0196-0709(80)80030-5 7446848

[9] Fraysse BG Alonso A House WF. Meniere's disease and endolymphatic hydrops: clinical-histopathological correlations. Ann Otol Rhinol Laryngol Suppl. (1980) 89:2–22. 10.1177/00034894800896S201 6779694

[10] Greco A Gallo A Fusconi M Marinelli C Macri GF Vincentiis Mde. Meniere's disease might be an autoimmune condition? Autoimmun Rev. (2012) 11:731–8. 10.1016/j.autrev.2012.01.004 22306860

[11] P. Gates. Hypothesis: could Meniere's disease be a channelopathy? Intern Med J. (2005) 35:488–9. 10.1111/j.1445-5994.2005.00891.x 16176473

[12] Nakashima T Naganawa S Sugiura M Teranishi M Sone M Hayashi H. Visualization of endolymphatic hydrops in patients with Meniere's disease. Laryngoscope. (2007) 117:415–20. 10.1097/MLG.0b013e31802c300c 17279053

[13] Naganawa S Yamazaki M Kawai H Bokura K Sone M Nakashima T. Visualization of endolymphatic hydrops in Meniere's disease with single-dose intravenous gadolinium-based contrast media using heavily T(2)-weighted 3D-FLAIR. Magn Reson Med Sci. (2010) 9:237–42. 10.2463/mrms.9.237 21187694

[14] Fang ZM Chen X Gu X Liu Y Zhang R Cao DR. A new magnetic resonance imaging scoring system for perilymphatic space appearance after intratympanic gadolinium injection, and its clinical application. J Laryngol Otol. (2012) 126:454–9. 10.1017/S0022215112000060 22314202

[15] Barath K Schuknecht B Naldi AM Schrepfer T Bockisch CJ Hegemann SC. Detection and grading of endolymphatic hydrops in Meniere disease using MR imaging. AJNR Am J Neuroradiol. (2014) 35:1387–92. 10.3174/ajnr.A3856 24524921 PMC7966587

[16] Naganawa S Satake H Kawamura M Fukatsu H Sone M Nakashima T. Separate visualization of endolymphatic space, perilymphatic space and bone by a single pulse sequence; 3D-inversion recovery imaging utilizing real reconstruction after intratympanic Gd-DTPA administration at 3 Tesla. Eur Radiol. (2008) 18:920–4. 10.1007/s00330-008-0854-8 18324405

[17] Naganawa S Yamazaki M Kawai H Bokura K Sone M Nakashima T. Imaging of Meniere's disease after intravenous administration of single-dose gadodiamide: utility of subtraction images with different inversion time. Magn Reson Med Sci. (2012) 11:213–9. 10.2463/mrms.11.213 23037568

[18] Naganawa S Suzuki K Nakamichi R Bokura K Yoshida T Sone M. Semi-quantification of endolymphatic size on MR imaging after intravenous injection of single-dose gadodiamide: comparison between two types of processing strategies. Magn Reson Med Sci. (2013) 12:261–9. 10.2463/mrms.2013-0019 24172793

[19] Cho YS Ahn JM Choi JE Park HW Kim YK Kim HJ. Usefulness of intravenous gadolinium inner ear MR imaging in diagnosis of Meniere's disease. Sci Rep. (2018) 8:17562. 10.1038/s41598-018-35709-5 30510158 PMC6277445

[20] Choi JE Kim YK Cho YS Lee K Park HW Yoon SH. Morphological correlation between caloric tests and vestibular hydrops in Meniere's disease using intravenous Gd enhanced inner ear MRI. PLoS ONE. (2017) 12:e0188301. 10.1371/journal.pone.0188301 29190293 PMC5708622

[21] Nakashima T Naganawa S Pyykkö I Gibson WP Sone M Nakata S. Grading of endolymphatic hydrops using magnetic resonance imaging. Acta Otolaryngol Suppl. (2009) 129:5–8. 10.1080/00016480902729827 19221900

[22] Naganawa S Kawai H Taoka T Sone M. Improved HYDROPS: imaging of endolymphatic hydrops after intravenous administration of gadolinium. Magn Reson Med Sci. (2017) 16:357–61. 10.2463/mrms.tn.2016-0126 28529249 PMC5743528

[23] Furman JM Jacob RG. Jongkees' formula re-evaluated: order effects in the response to alternate binaural bithermal caloric stimulation using closed-loop irrigation. Acta Otolaryngol. (1993) 113:3–10. 10.3109/00016489309135759 8442419

[24] Morimoto K Yoshida T Sugiura S Kato M Kato K Teranishi M. Endolymphatic hydrops in patients with unilateral and bilateral Meniere's disease. Acta Otolaryngol. (2016) 137:23–8. 10.1080/00016489.2016.1217042 27564645

[25] Wu Q Dai C Zhao M Sha Y. The correlation between symptoms of definite Meniere's disease and endolymphatic hydrops visualized by magnetic resonance imaging. Laryngoscope. (2016) 126:974–9. 10.1002/lary.25576 26333096

[26] Lin MY Timmer FC Oriel BS Zhou G Guinan JJ Kujawa SG. Vestibular evoked myogenic potentials (VEMP) can detect asymptomatic saccular hydrops. Laryngoscope. (2006) 116:987–92. 10.1097/01.mlg.0000216815.75512.03 16735912 PMC2758415

[27] Lopez-Escamez JA Carey J Chung WH Goebel JA Magnusson M Mandala M. Equilibrium Committee of the American Academy of, S Neck, and S Korean Balance, Diagnostic criteria for Meniere's disease. J Vestib Res. (2015) 25:1–7. 10.3233/VES-150549 25882471

[28] Shiraishi K Ohira N Kobayashi T Sato M Osaki Y Doi K. Comparison of furosemide-loading cervical vestibular-evoked myogenic potentials with magnetic resonance imaging for the evaluation of endolymphatic hydrops. Acta Otolaryngol. (2020) 140:723–7. 10.1080/00016489.2020.1769863 32700983

[29] Murofushi T Tsubota M Kanai Y Endo H Ushio M. Association of cervical vestibular-evoked myogenic potential tuning property test results with MRI findings of endolymphatic hydrops in Meniere's disease. Eur Arch Otorhinolaryngol. (2021) 278:3267–73. 10.1007/s00405-020-06410-z 33037440

[30] Sluydts M Bernaerts A Casselman JW Foer BDe Blaivie C Zarowski A. The relationship between cochleovestibular function tests and endolymphatic hydrops grading on MRI in patients with Meniere's disease. Eur Arch Otorhinolaryngol. (2021) 278:4783–93. 10.1007/s00405-021-06610-1 33492418

[31] He J Peng A Hu J Zhang Z Chen Y Wang Q. Dynamics in endolymphatic hydrops & symptoms in Meniere's disease after endolymphatic duct blockage, preliminary results. Front Neurol. (2020) 11:622760. 10.3389/fneur.2020.622760 33551977 PMC7859097

[32] Zhang W Hui L Zhang B Ren L Zhu J Wang F. The correlation between endolymphatic hydrops and clinical features of Meniere disease. Laryngoscope. (2021) 131:E144–50. 10.1002/lary.28576 32083730

[33] Xie J Zhang W Zhu J Hui L Li S Ren L. Differential diagnosis of endolymphatic hydrops between “Probable” and “Definite” Meniere's Disease *via* magnetic resonance imaging. Otolaryngol Head Neck Surg. (2021) 165:696–700. 10.1177/0194599821990680 33528304

[34] Ishiyama G Lopez IA Sepahdari AR Ishiyama A. Meniere's disease: histopathology, cytochemistry, and imaging. Ann N Y Acad Sci. (2015) 1343:49–57. 10.1111/nyas.12699 25766597

[35] Schunknecht HF Montandon P. Pathology of the ear in pneumococcal meningitis. Arch Klin Exp Ohren Nasen Kehlkopfheilkd. (1970) 195:207–25. 10.1007/BF00302950 5435957

[36] Nam SI. Endolymphatic Hydrops: Pathophysiology and Etiology. Korean J Otorhinolaryngol Head Neck Surg. (2011) 54:509–518. 10.3342/kjorl-hns.2011.54.8.509

[37] Wit HP Warmerdam TJ Albers FW. Measurement of the mechanical compliance of the endolymphatic compartments in the guinea pig. Hear Res. (2000) 145:82–90. 10.1016/S0378-5955(00)00078-2 10867280

[38] Salvinelli F Greco F Trivelli M Linthicum FH Jr. Meniere's disease Histopathological changes: a post mortem study on temporal bones. Eur Rev Med Pharmacol Sci. (1999) 3:189–93. 11073127

[39] Cho YS Cho K Park CJ Chung MJ Kim JH Kim K. Automated measurement of hydrops ratio from MRI in patients with Meniere's disease using CNN-based segmentation. Sci Rep. (2020) 10:7003. 10.1038/s41598-020-63887-8 32332804 PMC7181627

[40] Kahn L Hautefort C Guichard JP Toupet M Jourdaine C Vitaux H. Relationship between video head impulse test, ocular and cervical vestibular evoked myogenic potentials, and compartmental magnetic resonance imaging classification in Meniere's disease. Laryngoscope. (2020) 130:E444–52. 10.1002/lary.28362 31742710

[41] Bernaerts A Vanspauwen R Blaivie C van Dinther J Zarowski A Wuyts FL. The value of four stage vestibular hydrops grading and asymmetric perilymphatic enhancement in the diagnosis of Meniere's disease on MRI. Neuroradiology. (2019) 61:421–9. 10.1007/s00234-019-02155-7 30719545 PMC6431299

