# Supplementary material for: Correlation of clinical parameters with endolymphatic hydrops on MRI in Meniere's disease
Source: Front Neurol. 2022 Jul 25;13:937703. doi: 10.3389/fneur.2022.937703 (PMC9361122; doi:10.3389/fneur.2022.937703)
Supplement: Supplementary file 1 [file Data_Sheet_1.docx]

Supplementary Material

**Supplementary Table 1** Number and percent of ears with cochlear and vestibular hydrops grades 0–2 according to the Meniere’s disease diagnosis (unaffected, possible, probable, or definite)

|  | Grade 0  n (%) | Grade 1  n (%) | Grade 2  n (%) |
| --- | --- | --- | --- |
| Cochlear hydrops |  |  |  |
| Unaffected (n = 117) | 113 (96.6%) | 4 (3.4%) | 0 (0%) |
| Possible (n = 37) | 35 (94.6%) | 1 (2.7%) | 1 (2.7%) |
| Probable (n = 12) | 8 (66.7%) | 4 (33.3%) | 0 (0%) |
| Definite (n = 80) | 34 (42.5%) | 33 (41.25%) | 13 (16.25%) |
| Vestibular hydrops |  |  |  |
| Unaffected (n = 117) | 102 (87.2%) | 14 (12.0%) | 1 (0.8%) |
| Possible (n = 37) | 27 (73.0%) | 7 (18.9%) | 3 (8.1%) |
| Probable (n = 12) | 5 (41.7%) | 5 (41.7%) | 2 (16.6%) |
| Definite (n = 80) | 16 (20%) | 29 (36.25%) | 35 (43.75%) |

n = number of ears.

**Supplementary Table 2** Analysis of cochlear hydrops grade according to MD diagnosis (unaffected, possible, probable, or definite) in 246 ears

| MD classification | Adjusted p-value | Fischer p-value |
| --- | --- | --- |
| Unaffected : Possible | 0.310 | < 0.0001 |
| Unaffected : Probable | 0.0051 |  |
| Unaffected : Definite | < 0.0001 |  |
| Possible : Probable | 0.015 |  |
| Possible : Definite | < 0.0001 |  |
| Probable : Definite | 0.0269 |  |

Analysis was determined by Fisher’s exact test with pairwise Fisher’s exact tests as post hoc analysis.

**Supplementary Table 3** Analysis of vestibular hydrops grade according to MD diagnosis (unaffected, possible, probable, or definite) in 246 ears

| MD classification | Adjusted p-value | Fischer p-value |
| --- | --- | --- |
| Unaffected : Possible | 0.0483 | < 0.0001 |
| Unaffected : Probable | 0.000858 |  |
| Unaffected : Definite | < 0.0001 |  |
| Possible : Probable | 0.124 |  |
| Possible : Definite | < 0.0001 |  |
| Probable : Definite | 0.124 |  |

Analysis was determined by Fisher’s exact test with pairwise Fisher’s exact tests as post hoc analysis.

**Supplementary Table 4** Analysis of PTA and LTA according to cochlear hydrops grades (0–2) in 246 ears

| Cochlear hydrops grade | n1 | n2 | Audiometry Threshold (dB) 1 | Audiometry Threshold (dB) 2 | 95% confidence interval | Adjusted  p-value | ANOVA p-value |
| --- | --- | --- | --- | --- | --- | --- | --- |
| PTA |  |  |  |  |  |  |  |
| 0 vs. 1 | 190 | 42 | 26.23 | 54.05 | −37.09 to −18.54 | < 0.0001 | < 0.0001 |
| 0 vs. 2 | 190 | 14 | 26.23 | 69.64 | −58.48 to −28.35 | < 0.0001 |  |
| 1 vs. 2 | 42 | 14 | 54.05 | 69.64 | −32.38 to 1.19 | 0.075 |  |
| LTA |  |  |  |  |  |  |  |
| 0 vs. 1 | 190 | 42 | 22.48 | 56.55 | −42.92 to −25.21 | < 0.0001 | < 0.0001 |
| 0 vs. 2 | 190 | 14 | 22.48 | 67.14 | −59.03 to −30.29 | < 0.0001 |  |
| 1 vs. 2 | 42 | 14 | 56.55 | 67.14 | −26.61 to 5.42 | 0.267 |  |

Analysis was determined using one-way ANOVA followed by Scheffé’s post hoc multiple comparison test. n1 and n2: number of ears of each hydrops grade that were compared and analyzed.

**Supplementary Table 5** Analysis of PTA and LTA according to vestibular hydrops grades (0–2) in 246 ears

| Vestibular hydrops grade | n1 | n2 | Audiometry Threshold (dB) 1 | Audiometry Threshold (dB) 2 | 95% confidence interval | Adjusted  p-value | Welch’s ANOVA p-value |
| --- | --- | --- | --- | --- | --- | --- | --- |
| PTA |  |  |  |  |  |  |  |
| 0 vs. 1 | 150 | 55 | 22.04 | 45.11 | −32.72 to −13.43 | < 0.0001 | < 0.0001 |
| 0 vs. 2 | 150 | 41 | 22.04 | 59.54 | −46.60 to −28.40 | < 0.0001 |  |
| 1 vs. 2 | 55 | 41 | 45.11 | 59.54 | −26.63 to −2.23 | 0.016 |  |
| LTA |  |  |  |  |  |  |  |
| 0 vs. 1 | 150 | 55 | 18.62 | 43.09 | −34.10 to −14.84 | < 0.0001 | < 0.0001 |
| 0 vs. 2 | 150 | 41 | 18.62 | 59.11 | −49.90 to −31.07 | < 0.0001 |  |
| 1 vs. 2 | 55 | 41 | 43.09 | 59.11 | −28.60 to −3.44 | 0.009 |  |

Analysis was determined using Welch’s ANOVA followed by Games-Howell’s post hoc multiple comparison test. n1 and n2: number of ears of each hydrops grade that were compared and analyzed.

**Supplementary Table 6** Analysis of PTA and LTA according to hydrops grades (0–2) in the cochlea in 80 ears with definite MD

| Cochlear hydrops grade | n1 | n2 | Audiometry Threshold (dB) 1 | Audiometry Threshold (dB) 2 | 95% confidence interval | Adjusted  p-value | ANOVA p-value |
| --- | --- | --- | --- | --- | --- | --- | --- |
| PTA |  |  |  |  |  |  |  |
| 0 vs. 1 | 34 | 33 | 49.78 | 54.43 | −19.16 to 9.86 | 0.727 | 0.048 |
| 0 vs. 2 | 34 | 13 | 49.78 | 69.23 | −38.82 to −0.09 | 0.049 |  |
| 1 vs. 2 | 33 | 13 | 54.43 | 69.23 | −34.25 to 4.65 | 0.171 |  |
| LTA |  |  |  |  |  |  |  |
| 0 vs. 1 | 34 | 33 | 44.75 | 57.42 | −27.57 to 2.24 | 0.112 | 0.014 |
| 0 vs. 2 | 34 | 13 | 44.75 | 66.67 | −41.80 to −2.02 | 0.027 |  |
| 1 vs. 2 | 33 | 13 | 57.42 | 66.67 | −29.22 to 10.73 | 0.516 |  |

Analysis was determined by one-way ANOVA with Scheffé’s post hoc multiple comparison test. n1 and n2: number of ears of each hydrops grade that were compared and analyzed.

**Supplementary Table 7** Analysis of PTA and LTA according to hydrops grades (0–2) in the vestibule in 80 ears with definite MD

| Vestibular hydrops grade | n1 | n2 | Audiometry Threshold (dB) 1 | Audiometry Threshold (dB) 2 | 95% confidence interval | Adjusted  p-value | ANOVA p-value |
| --- | --- | --- | --- | --- | --- | --- | --- |
| PTA |  |  |  |  |  |  |  |
| 0 vs. 1 | 16 | 29 | 41.33 | 55.3 | −32.35 to 4.40 | 0.172 | 0.03 |
| 0 vs. 2 | 16 | 35 | 41.33 | 60.68 | −37.16 to −1.54 | 0.030 |  |
| 1 vs. 2 | 29 | 35 | 55.3 | 60.68 | −20.19 to 9.44 | 0.665 |  |
| LTA |  |  |  |  |  |  |  |
| 0 vs. 1 | 16 | 29 | 36.88 | 54.94 | −36.94 to 0.80 | 0.064 | 0.009 |
| 0 vs. 2 | 16 | 35 | 36.88 | 60 | −41.41 to −4.84 | 0.009 |  |
| 1 vs. 2 | 29 | 35 | 54.94 | 60 | −20.27 to 10.16 | 0.710 |  |

Analysis was determined by one-way ANOVA with Scheffé’s post hoc multiple comparison test. n1 and n2: number of ears of each hydrops grade that were compared and analyzed.

**Supplementary Table 8** Analysis of CP according to the cochlear and vestibular hydrops grade (0–2) in 107 ears with MD

| Hydrops  Grade | n1 | n2 | CP (%) 1 | CP (%) 2 | 95% confidence interval | Adjusted  p-value | ANOVA p-value | |
| --- | --- | --- | --- | --- | --- | --- | --- | --- |
| Cochlear |  |  |  |  |  |  |  |  |
| 0 vs. 1 | 58 | 36 | 22.95 | 37.42 | −28.69 to −0.25 | 0.045 | 0.010 | |
| 0 vs. 2 | 58 | 13 | 22.95 | 42.93 | −40.56 to −0.59 | 0.059 |  |  |
| 1 vs. 2 | 36 | 13 | 37.42 | 42.93 | −27.21 to 16.18 | 0.820 |  |  |
| Vestibular |  |  |  |  |  |  |  | |
| 0 vs. 1 | 34 | 35 | 20.64 | 26.68 | −21.99 to 9.89 | 0.643 | 0.003 | |
| 0 vs. 2 | 34 | 38 | 20.64 | 42.12 | −37.10 to −5.85 | 0.004 |  |  |
| 1 vs. 2 | 35 | 38 | 26.68 | 42.12 | −30.94 to −0.08 | 0.051 |  |  |

Analysis was determined by one-way ANOVA with Scheffé’s post hoc multiple comparison test. n1 and n2: number of ears for each hydrops grade that were compared and analyzed.

**Supplementary Table 9** Associations of cochlear and vestibular hydrops (grades 0–2) with disease duration in 123 ears of 123 patients

| Hydrops  Grade | n1 | n2 | Duration (years) 1 | Duration (years) 2 | ANOVA  p-value |
| --- | --- | --- | --- | --- | --- |
| Cochlear |  |  |  |  |  |
| 0 vs. 1 | 71 | 38 | 4.077 | 4.057 | 0.997 |
| 0 vs. 2 | 71 | 14 | 4.077 | 4.132 |  |
| 1 vs. 2 | 38 | 14 | 4.057 | 4.132 |  |
| Vestibular |  |  |  |  |  |
| 0 vs. 1 | 43 | 40 | 3.265 | 4.494 | 0.135 |
| 0 vs. 2 | 43 | 40 | 3.265 | 4.534 |  |
| 1 vs. 2 | 40 | 40 | 4.494 | 4.534 |  |

Analysis was determined by one-way ANOVA with Scheffé’s post hoc multiple comparison test. n1 and n2: number of ears of each hydrops grade that were compared and analyzed.

**Supplementary Figure 1.** Associations of cochlear **(A)** and vestibular **(B)** hydrops grades 0, 1, and 2 with disease duration in 123 patients (123 ears). The associations for both types of hydrops were not statistically significant.
