# Supplementary figures and images for: Correlation of clinical parameters with endolymphatic hydrops on MRI in Meniere's disease
Source: Front Neurol. 2022 Jul 25;13:937703. doi: 10.3389/fneur.2022.937703 (PMC9361122; doi:10.3389/fneur.2022.937703)

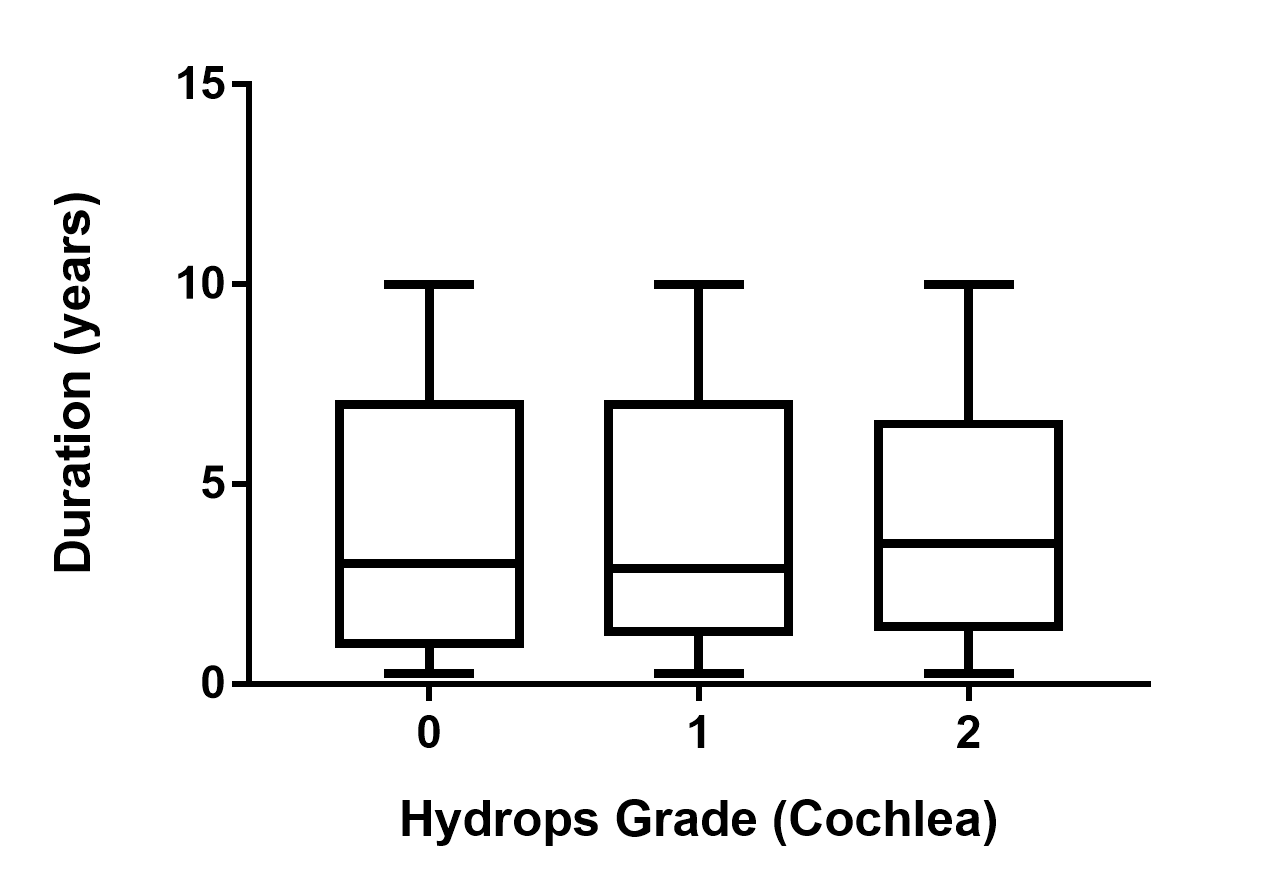

Supplement: Supplementary file 2 [file Image_1.TIF]

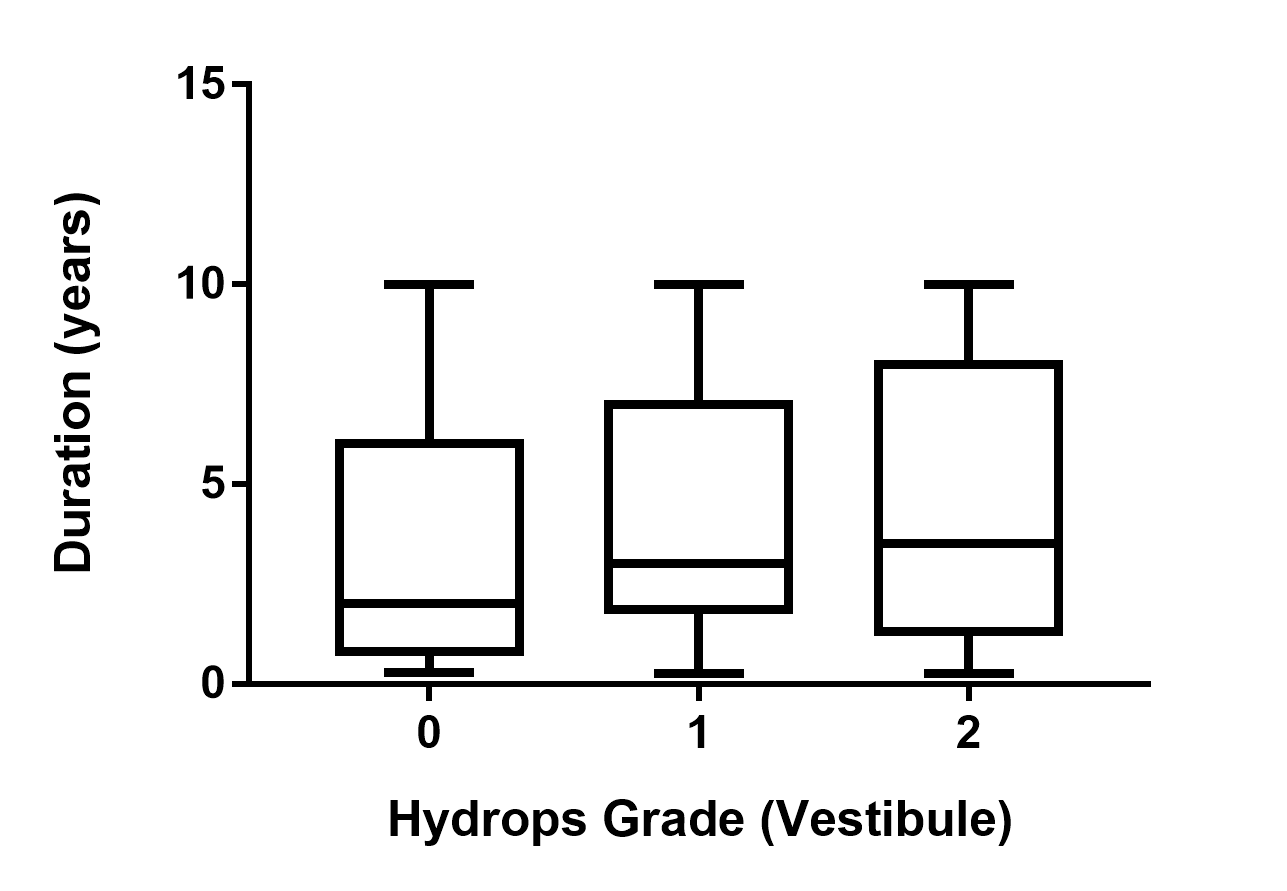

Supplement: Supplementary file 3 [file Image_2.TIF]
